# Evolutionary Genomics Reveals Lineage-Specific Gene Loss and Rapid Evolution of a Sperm-Specific Ion Channel Complex: CatSpers and CatSperβ

**DOI:** 10.1371/journal.pone.0003569

**Published:** 2008-10-30

**Authors:** Xinjiang Cai, David E. Clapham

**Affiliations:** 1 Department of Cell Biology, Duke University Medical Center, Durham, North Carolina, United States of America; 2 Department of Medicine (Cardiology), Duke University Medical Center, Durham, North Carolina, United States of America; 3 Howard Hughes Medical Institute, Department of Cardiology, Children's Hospital, and Department of Neurobiology, Harvard Medical School, Boston, Massachusetts, United States of America; University of Cincinnati, United States of America

## Abstract

The mammalian CatSper ion channel family consists of four sperm-specific voltage-gated Ca^2+^ channels that are crucial for sperm hyperactivation and male fertility. All four CatSper subunits are believed to assemble into a heteromultimeric channel complex, together with an auxiliary subunit, CatSperβ. Here, we report a comprehensive comparative genomics study and evolutionary analysis of CatSpers and CatSperβ, with important correlation to physiological significance of molecular evolution of the CatSper channel complex. The development of the CatSper channel complex with four CatSpers and CatSperβ originated as early as primitive metazoans such as the Cnidarian *Nematostella vectensis*. Comparative genomics revealed extensive lineage-specific gene loss of all four CatSpers and CatSperβ through metazoan evolution, especially in vertebrates. The CatSper channel complex underwent rapid evolution and functional divergence, while distinct evolutionary constraints appear to have acted on different domains and specific sites of the four CatSper genes. These results reveal unique evolutionary characteristics of sperm-specific Ca^2+^ channels and their adaptation to sperm biology through metazoan evolution.

## Introduction

In spermatozoa, Ca^2+^ influxes through plasma membrane Ca^2+^ channels play a key role in mediating sperm maturation, motility, and the acrosome reaction [1]–[3]. Sperm motility is driven by flagellar ATP-dependent dynein motor proteins. Basic flagellar architecture is conserved across species; flagellar movement does not require calcium, or even a plasma membrane. In general, sperm acquire motility after they exit the male reproductive system.

Both initiation of motility and modulation of motility vary in a species-specific fashion. Initiation of sperm motility display many features distinct from modulation of motility. In many mammals, motility is characterized by symmetrical movement of the tail and progressive movement (normal swimming), but the initiating signal or signals are poorly understood. Hyperactivated motility in mammals occurs later, as the sperms encounter progressively more alkaline environments as they ascend the reproductive tract. The primary characteristic of sperm hyperactivated motility in well-characterized mammalian species is a large bend angle between head and tail [4], [5]. This larger sweep of the tail results in substantially more force than the force of sperm cells swimming before hyperactivation [6]. This force has been proposed to free sperm from surfaces or trapping spaces in the uterus and oviduct, and/or penetrate the cumulus and the thick protective wall of the zona pellucida [7]. In sea urchin, the chemoattractant peptide, Resact, initiates an increase in intracellular [Ca^2+^] and change in the bend angle, resulting in larger swimming arcs [8]. In contrast, marine fish spermatozoa motility [9] is initiated by the large change in osmolality as they exit the fish and encounter seawater, but this initiation of motility is not Ca^2+^-dependent [10]. These marine teleosts have up to 10 times the beat frequency of mammalian sperm cells, but swim only for short distances to eggs deposited in seawater [9]. A change in flagellar bend angle, equivalent to hyperactivation in mammals, has not been reported, and indeed may not be required for sperm docking to the marine fish egg pyle. In birds and reptiles, sperm are often stored in specialized tubules connected to the oviduct. In chickens, initiation, or re-initiation of motility may be related to temperature sensitivity of a plasma membrane Ca^2+^ ATPase [11]. Thus, although all spermatozoa have the basic ATP-dynein motor required for normal swimming, they appear to differ in modulation of motility in a species-specific manner.

Several types of Ca^2+^-permeant channel proteins, such as voltage-gated Ca^2+^ (Ca_V_), CatSper, cyclic nucleotide-gated (CNG), and Transient Receptor Potential (TRP) channels, have been detected in spermatocytes or spermatozoa [1]–[3], [12], but until recently their function could only be assayed in spermatocytes. Among the Ca^2+^-permeant channels, CatSper channels mediate the dominant Ca^2+^-carrying current in mouse epididymal spermatozoa and induce sperm hyperactivation during sperm capacitation [13]. Intracellular alkalinization triggers CatSper activation and subsequent hyperactivation of motility in mice. In addition, in the absence of CatSper current, sperm cell motility endurance declines [12]–[15].

The mammalian CatSper family of ion channels is composed of four members, CatSper1-4 [14], [16]–[19]. All four CatSper proteins are expressed in sperm cells and functionally localized to the principal piece of the sperm tail [12]. Like most of the voltage-gated ion channels [20], the six-transmembrane-spanning (6-TMS) CatSpers are believed to form a tetrameric structure. All four CatSpers seem to be required to mediate functional alkalinization-activated Ca^2+^-selective sperm currents [12]. Targeted deletion of any of the four *CatSper* genes results in mouse male infertility and an identical sperm cell phenotype including loss of sperm hyperactivation [12], [14], [21]–[23], while mice lacking other ion channel genes, if viable, are fertile [1]. Interestingly, a sperm-specific two-TMS protein, CatSperβ, is associated with the CatSper1 channel subunit in mouse testis [24]. CatSperβ displays a similar localization pattern with CatSpers, copurifies with CatSper proteins, and is absent in *CatSper1* ^−/−^ sperm. Thus, CatSperβ is an auxiliary subunit of the CatSper channel complex [24], but its role in channel activity is unknown.

In the absence of suitable heterologous expression systems, our current understanding of the physiological role of CatSpers is primarily based on studies using mouse models [12]–[14], [21]–[23]. Reproductive proteins usually undergo rapid evolution and functional divergence because selective evolutionary forces such as sperm competition, sexual selection and sexual conflict may act on reproductive proteins to generate phenotypic diversity through speciation [25]–[27]. In addition, comparative genomics studies and evolutionary analyses can provide insights into molecular, biophysical, and biochemical analyses of functional and regulatory mechanisms of ion channels and transporters [28]–[33]. Therefore, to better understand the functional role of the CatSper channel complex, we set out to determine the evolutionary origins and patterns of CatSpers and CatSperβ using extensive database mining and rigorous phylogenetic analyses, and to analyze the evolutionary rates and functional divergence of CatSpers and CatSperβ.

## Materials and Methods

### Database mining, sequence alignment and phylogenetic analysis

TBlastN and BlastP searches [34] using protein sequences of *Homo sapiens* CatSper and CatSperβ protein sequences were performed on the genomic and protein databases of the National Center for Biotechnology Information (NCBI) (http://www.ncbi.nlm.nih.gov/blast/), Ensembl (http://www.ensembl.org/Multi/blastview), and the Joint Genome Institute (JGI) (http://www.jgi.doe.gov/). *H. sapiens* and *Nematostella vectensis* CatSper and CatSperβ sequences were also used for BlastN and TBlastN searches of three sponge databases - *Amphimedon queenslandica* genomic traces at NCBI, SpongeBase (http://spongebase.uni-mainz.de/), and *Oscarella carmela* EST database [35]. TBlastN searches were also conducted on the survey genomic database of the elephant shark, *Callorhinchus milii* (http://esharkgenome.imcb.a-star.edu.sg/). Protein sequences of the bacterial Na_V_ channel superfamily were obtained from the bacterial protein cluster CLS1187052 at the NCBI protein cluster database and sequences characterized previously [36].

Sequence alignments, manual editing, and phylogenetic analysis for the collected dataset were carried out essentially as previously described [29], [32], [37].

### Chromosome synteny

The Evolutionarily Conserved Regions (ECR) browser (http://ecrbrowser.dcode.org/) was used for initial screening of syntenic chromosomal regions among *H. sapiens*, *Mus musculus*, *Gallus gallus*, *Fugu rubripes*, and *Xenopus tropicalis* genomes. However, only *M. musculus* and *G. gallus* genomes showed substantial synteny in *H. sapiens* chromosomal regions flanking the *CatSpers* and *CatSperβ* genes, and were used for further analysis. The flanking orthologous genes were identified on the ECR browser and/or the NCBI Map Viewer (http://www.ncbi.nlm.nih.gov/mapview/).

### Estimation of non-synonymous (d_N_) to synonymous (d_S_) nucleotide substitution ratio

*H. sapiens* and *M. musculus* mRNA sequences of *CatSper* and *CatSperβ* genes were retrieved from the NCBI database, and then converted to codon alignments by the PAL2NAL server [38], using corresponding protein sequence alignments. *d_N_* and *d_S_* values were calculated with the codeml program implemented in the PAML package [39].

## Results and Discussion

### Development of four distinct CatSper genes and CatSperβ in early Eumetazoa

**Figure 1:**
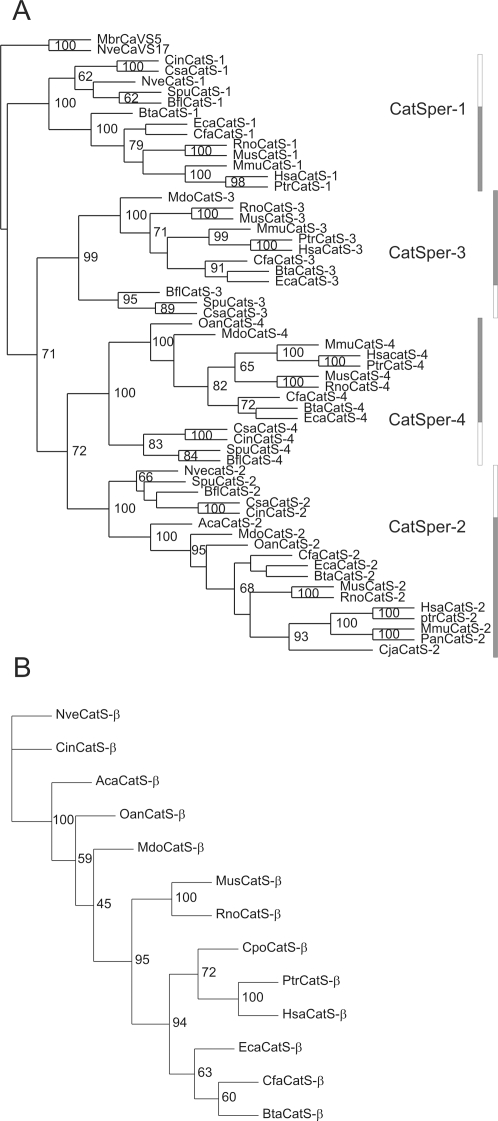
Phylogenetic reconstruction of the evolutionary history of CatSpers and CatSperβ in metazoans. The phylogenetic trees of the CatSper protein family (*A*) and the CatSperβ protein family (*B*) were constructed using the maximum likelihood approach [37]. Two putative primitive Ca^2+^ channels (MbrCa_V_S5 and NveCa_V_S17) were used as the outgroup for the CatSper family. Bootstrap values of more than 60 are shown at corresponding branches. The CatSper1-4 groups are indicated by rectangular bars, with invertebrate CatSpers filled with *white* and vertebrate CatSpers with *gray*. Note that protein sequences that failed in the chi-square test in Tree-Puzzle [69] or contained more than 15% gaps in the refined alignments were not subjected to phylogenetic analysis (Table S1). Abbreviations used: Aca, *A. carolinensis*; Bfl, *B. floridae*; Bta, *B. taurus*; Cfa, *C. familiaris*; Cin, *C. intestinalis*; Cja, *C. jacchus*; Cpo, *C. porcellus*; Csa, *C. savignyi*; Eca, *E. caballus*; Hsa, *H. sapiens*; Mdo, *M. domestica*; Mmu, *M. mulatta*; Mus, *M. musculus*; Nve, *N. vectensis*; Oan, *O. anatinus*; Pan, *P. anubis*; Ptr, *P. troglodytes*; Rno, *R. norvegicus*; Spu, *S. purpuratus*.

Mouse gene knockout studies indicate that all four CatSper subunits are required to mediate functional Ca^2+^-selective sperm currents necessary for sperm hyperactivation [12]–[14], [21]. However, previous reports have suggested the presence of fewer copies of CatSper homologues in early deuterostomes: three in *Ciona intestinalis* [40] and two in sea urchin testis [24]. Here, extensive genomic analysis of two sea squirts, *C. intestinalis* and *Ciona savignyi*, the sea urchin, *Strongylocentrotus purpuratus*, and the most basal extant chordate lineage, the amphioxus *Branchiostoma floridae* [41], [42], demonstrated the presence of four CatSper subunits and single copies of CatSperβ in these four species (Fig. 1A and Supplementary Table S1). Therefore, the CatSper channel complex containing four CatSper subunits and one CatSperβ had developed in early deuterostomes.

As one of the unicellular ancestors of Metazoa [43], choanoflagellates possess homologues of many components of animal Ca^2+^ signaling and amplification pathways (the Ca^2+^ signaling ‘toolkit’ [44]) but not CatSper channels [33]. To further determine the evolutionary origins of CatSper channels, we next searched the currently available databases of metazoans that branched off the metazoan stem before the radiation of bilaterians – the genome sequences of Cnidarian *Nematostella vectensis* [45] and Placozoa *Trichoplax adhaerens* at JGI, the EST database of Ctenophora *Pleurobrachia pileus*, and EST and genomic trace databases of ancestral metazoan Porifera sponges.

Indeed, *N. vectensis* has four CatSper gene homologues, which can be classified into CatSper1-4 based on phylogenetic analysis, and a single homolog of CatSperβ (Fig. 1A and Table S1). Three EST sequences were also identified in the *P. pileus* EST database, which were further categorized as CatSper1 and CatSper2 (Table S1). No significant hits were found in the sponge EST and genomic trace databases and in the Placozoa genome. It should be noted that the negative results from currently available sponge databases should be viewed as provisional until the complete genome or sperm EST sequences from sponges have been examined.

**Figure 2:**
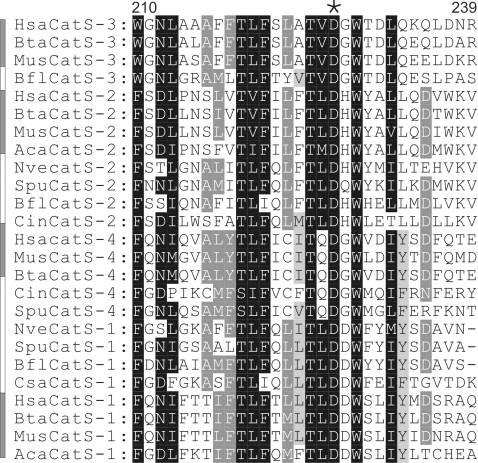
Conservation of the putative pore domain of CatSpers from invertebrate and vertebrate species. The putative pore regions of CatSpers from selected species, corresponding to amino acids 210–239 of human CatSper3 (GenBank Accession No., NP_821138.1), were aligned and manually edited to improve alignments. Invertebrate CatSpers are indicated with *white* rectangular bars and vertebrate CatSpers with *gray* rectangular bars. The key aspartate residue of CatSpers is overlined with an *asterisk* symbol. For abbreviations, please refer to the Fig. 1 legend.

The transmembrane segments (TMS) and the putative pore region of human and mouse CatSpers are highly conserved [17]. Sequence alignment of CatSper sequences identified in this study showed similar observations across invertebrate and vertebrate species (Fig. 2). The key aspartate residue of CatSpers, presumably corresponding to the highly conserved glutamate residues (or occasionally aspartate residues) in the pore loop regions that underlie Ca^2+^ selectivity in many Ca_V_ channels [46], is absolutely conserved (Fig. 2).

The identification of four CatSper homologues and CatSperβ in Cnidarians, but not in choanoflagellates, suggests that the origin of the CatSper complex could be dated back to early Eumetazoa, much earlier than previously thought [24]. Many other ion channels and transporters usually underwent further expansion in basal vertebrates after divergence from Urochordata [29], [31], [32], [47], [48]. However, the CatSper channel complex appears to be preserved from early metazoans to vertebrates without any further duplication events. The stringent requirement for four different CatSpers to form a putative heterotetrameric channel complex might render any further gene duplication of CatSpers excessive and the gene then be degenerated. For instance, a recent primate-specific duplication of *CatSper2* resulted in a *CatSper2* pseudogene (Table S4).

### Lineage-specific gene loss of CatSper and CatSperβ across the metazoan evolution

Sperm cells express a variety of Ca^2+^ channels and transporters [2], the majority of which are also expressed in other tissues and are often highly conserved from early metazoans to mammals, such as TRP channels [49], [50], Na^+^/Ca^2+^ exchangers (NCX) [29], [48], and sarco/endoplasmic reticulum Ca^2+^ ATPases [51], [52]. In contrast, little is known about the detailed evolutionary pattern of sperm-specific ion channels.

**Figure 3:**
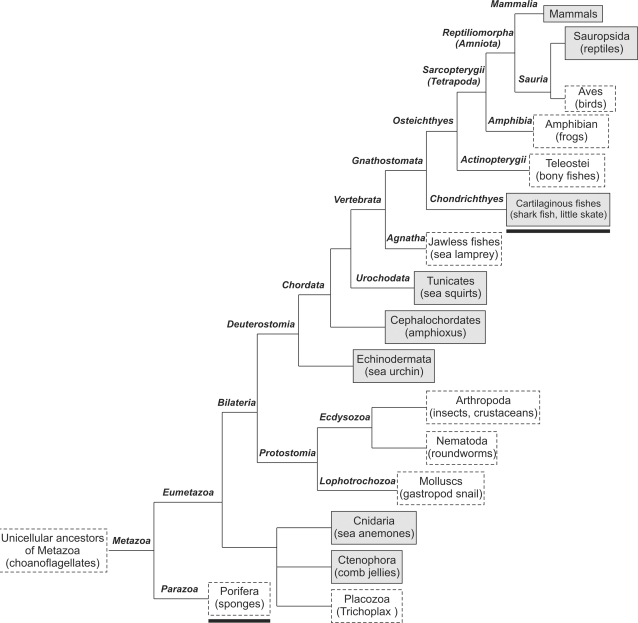
The metazoan phylogeny describing the presence or absence of CatSpers and CatSperβ in examined metazoan genomes. The phylogenetic branching patterns of metazoans (not proportional to the evolutionary rates) were extracted from the Tree of Life project (http://www.tolweb.org/tree/) as of July 30, 2008. The metazoan lineages known to contain the CatSper channel complex are indicated by boxes with a solid line and gray background, while those believed to lack the channel complex are marked by boxes with a broken line and white background. Two lineages (Porifera and cartilaginous fishes) are underlined since the results obtained from these lineages are based solely on survey genome or genomic traces rather than complete genome sequences. The availability of more metazoan genome sequences in the future will probably expand and/or refine the lineage branches shown here.

Fig. 3 shows the comprehensive evolutionary genomics of the CatSper ion channel complex. We have identified novel CatSper sequences from non-mammal vertebrates, such as the Anole lizard *Anolis carolinensis*, and cartilaginous fishes *Callorhinchus milii* and *Leucoraja erinacea* (Table S1). The CatSper channel complex is completely absent from a diverse sampling of protostome genomes. Interestingly, even though they are present in basal Chordata (amphioxus and sea squirts), all four CatSpers and CatSperβ appear to be lacking in several vertebrate lineages such as Agnatha (jawless fishes), Teleostei (bony fishes), Amphibia (frogs), and Aves (birds). This distinctive lineage distribution pattern of the CatSper channel complex (Fig. 3) is in drastic contrast to that of most ion channels and transporters characterized previously, which are usually highly conserved in metazoans [29], [31], [32], [40], [47], [48].

Two main evolutionary mechanisms might account for the intermittent pattern of presence or absence of genes in the genomes: lineage-specific gene loss and horizontal gene transfer [53], [54]. A bacterial voltage-gated Na^+^ channel, Na_V_BP, is believed to have the closest relationship with CatSpers, before the mammalian Ca_V_ and Na_V_ channel classes [19]. Similar to CatSpers, Na_V_BP is activated by alkalinization and is important for motility [55]. Horizontal gene transfer between prokaryotes and metazoans is not common, but possible [56]. Perhaps during the process of metazoan evolution, a bacterial channel protein like Na_V_BP became incorporated into sperm cells of some, but not all metazoan genomes. Such a bacterial channel would have undergone further functional divergence to enhance sperm motility by inducing Ca^2+^-dependent hyperactivation and subsequent species-dependent adaptation to reflect phylogeny. Alternatively, the absence of the CatSper complex in the specific metazoan lineages described above could be explained as a result of differential gene loss. Thus, in sperm cells of those metazoan lineages, Ca^2+^ influx across plasma membrane might not be important for sperm motility endurance or hyperactivated motility. Of course, other types of Ca^2+^ channels/sources might have substituted for the CatSper channel complex.

If lineage-specific gene loss had occurred, it might first render the gene degenerate and non-functional. Subsequent neutral evolution could then mask a substantial portion of the gene sequence, but short fragments in the coding sequence of the degenerated gene might still be present, with flanking functional genes on the syntenic chromosomes conserved across closely related species. Therefore, we compared syntenic chromosomal regions containing *CatSper* and *CatSperβ* genes in mouse and human genomes and those in bird, amphibian, and bony fish genomes.

**Figure 4:**
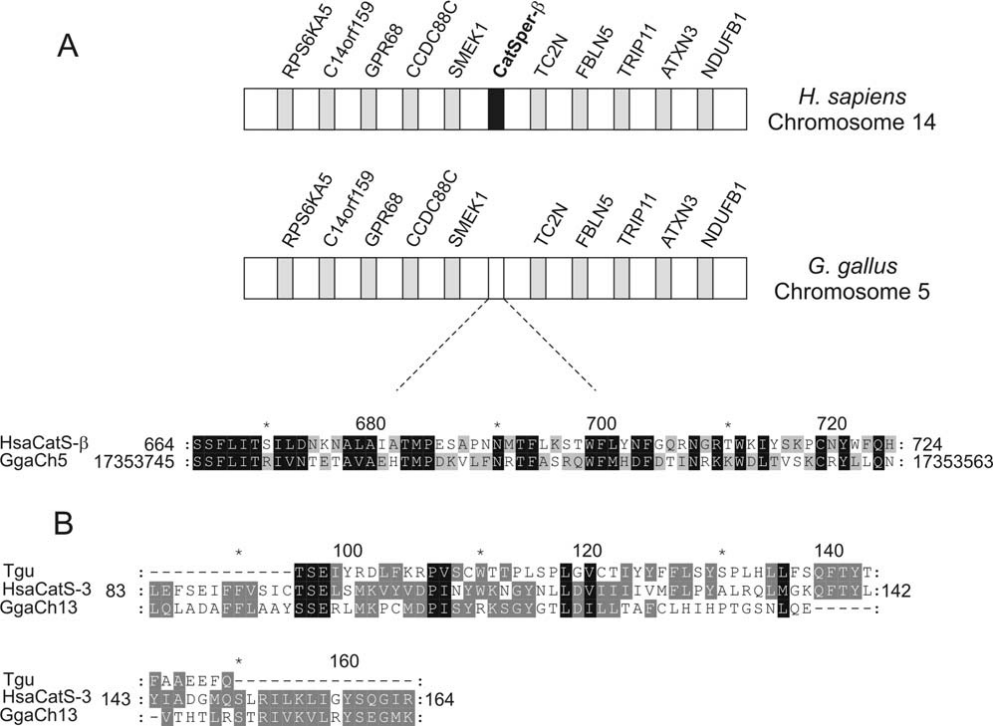
Chromosomal synteny between human and chicken genomes and sequence alignment of degenerated CatSperβ and CatSper3 fragments from bird genomes. *A,* chromosomal regions harboring degenerate DNA fragment of putative *CatSperβ* on chicken chromosome 5 with synteny to human chromosome 14. Shown here are ten genes flanking *H. sapiens CatSperβ* on human chromosome 14 and syntenic genes on chicken chromosome 5 (not to the scale of base-pair length). *CatSperβ* is indicated with a black rectangle and other genes with gray rectangles. The location of the degenerate genomic fragment of putative chicken *CatSperβ* is specified with an open rectangle, and the translated sequence is aligned with HsaCatSperβ. *B, H. sapiens* CatSper3 is aligned with the short stretch of sequences translated from putative degenerate chicken and zebra finch *CatSper3* fragments. Abbreviations for flanking genes can be found in Table S2.

By examining genomic regions with the ECR browser and the NCBI genomic database, we found highly conserved synteny between selected regions of the mouse and human genomes and those of the chicken genome for *CatSper2-4* and *CatSperβ* (Fig. 4 and Table S2, S3, S4, S5, S6). As shown in Fig. 4A and Table S2, 10 genes flanking the 5′- and 3′-end of *CatSperβ* on human chromosome 14 and corresponding orthologous genes on chicken chromosome 5 are syntenic. By using three different gene-finding programs [37], subsequent examination of chicken genomic sequence located between *SMEK1* and *TC2N* genes did not yield any obvious gene coding region, but a portion of the genomic sequence could be translated into a 66-aa fragment with high similarity to human CatSperβ (Fig. 4A) and other CatSperβ sequences (data not shown). Similar observations have also been observed for CatSper2 and CatSper3 (Tables S4 and S5). Thus, CatSper sequences might have been degenerated in chick genomes, with short fragments of coding sequences still present.

We next examined the preliminary genome sequence of the zebra finch *Taeniopygia guttata*. We also found a stretch of genomic sequence that could be translated into a short CatSper3 fragment sharing sequence similarity with a region in human CatSper3 and the putative chicken CatSper3 fragment (Fig. 4B). Such a short CatSper4 fragment was also made in the genome of the snail *Lottia gigantean* (data not shown). In addition, the EST libraries of a non-insect arthropod, the Arctic springtail *Onychiurus arcticus*, contain an EST clone (GenBank Accession No. EW749693.1) encoding a partial protein sequence in which the C-terminal (aa 68–218) displayed high similarity to TMS domains of CatSper4, with *N. vectensis* CatSper4 as the most significant hit (E-value, 3×e^−27^). The N-terminal 67 aa share no sequence homology to other CatSpers or known proteins.

Taken together, we conclude that the intermittent presence/absence pattern of the CatSper channel complex in metazoan genomes was likely derived from lineage-specific gene loss. Importantly, CatSperβ is present in genomes in which CatSpers are identified, but is missing in genomes that lack CatSpers. Thus, functional association between CatSperβ and CatSpers is not only critical for CatSperβ stability at the cellular level, i.e., mouse sperm cells [24], but might also relate to the all-or-none presence of CatSpers and CatSperβ at the genomic level.

Mouse spermatozoa have only two primary ion channel currents in normal saline solution, I_CatSper_ and I_Ksper_ [15], [57]. The Ca^2+^ current is mediated by CatSpers while the K^+^ current is mediated presumably by mSlo3. Both currents are effectively gated by alkalinization and voltage changes. Activated K^+^ current hyperpolarizes the flagellar plasma membrane and thus maximizes the entry of Ca^2+^ via Catsper by increasing driving force on Ca^2+^. In sea urchin, direct whole-sperm voltage clamp recordings have not been possible, but indirect evidence suggests that the chemoattractant peptide Resact activates a cyclic-nucleotide-gated (CNG) K^+^-selective conductance [8] (presumably, Sp-tetraKCNG channel [58]) . This K^+^ current may act in place of I_KSper_ to enhance Ca^2+^ entry. Mammalian spermatocytes may contain CNGB1 and perhaps CNGA3 channel proteins [59], but to date, currents consistent with CNG channels have not been observed in mouse sperm [57]. The simplest interpretation is that CatSpers are critical for the alteration of motility patterns for chemotaxis (sea urchin) and hyperactivation (mammals), but that the type of K channel activated (CNG or mSlo3) modulates CatSper's roles specific to the species. Thus, sea urchin eggs, with their thin walls, are easy for sperm to penetrate but sperm must be guided to the egg in the less restricted ocean environment by chemotactic peptide alteration of sea urchin sperm motility. In contrast, mouse spermatozoa require more force, and thus hyperactivated motility, to penetrate the thicker oocyte wall. Whether there is an additional chemotactic factor guiding spermatozoa in mammals is not established. Since birds and fish have thin oocyte cell walls, CatSper may have provided no evolutionary advantage and was degenerated in these species.

Such an extensive lineage-specific gene loss of an entire ion channel complex through metazoan evolution, especially in vertebrates, has not been documented in other channels and transporters. In some cases, such as for Na^+^/Ca^2+^ exchangers, one member, NCX4, which arose in basal vertebrates, persevered in teleost, amphibian and reptilian species but was lost in mammals and birds. Most of NCX members, NCX1-3, however, were still retained in all vertebrates examined [48]. Interestingly, a sperm-specific and unusual putative Na^+^/H^+^ exchanger required for sperm motility and fertility [60] also shows similar extensive lineage-specific gene loss in metazoans (Cai, X, unpublished observation). Thus, further evolutionary genomics studies of sperm-specific channels and transporters will no doubt shed novel insights into physiological roles of ion transport in sperm biology.

**Figure 5:**
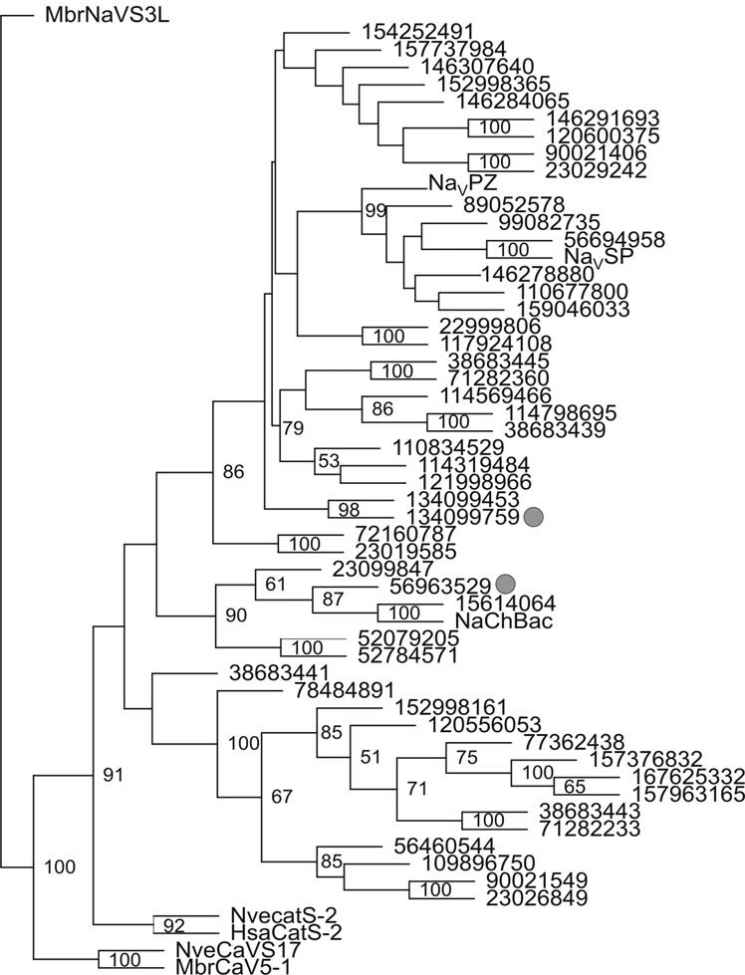
Phylogenetic tree of the 6-TMS bacterial ion channel family. A bootstrapped maximum parsimony tree was constructed with a Na_V_ channel homologue in choanoflagellates (*MbrNaV-S3L*) as an outgroup. Two CatSper sequences, *NveCatSper2* and *HsaCatSper2*, and two putative Ca_V_ channel homologues, *MbrCa_V_S5-1* and *NveCa_V_-S17*, are also included. The position of the key aspartate residue in the pore region of two bacterial proteins is marked by gray circles. Bootstrap values of >50 are shown at corresponding branches. Each branch of the tree is labeled with the GI numbers in the NCBI protein database for most organisms. NaChBac, Na_V_SP and Na_V_PZ channels were functionally characterized previously [36], [61]. Abbreviations: Hsa, *H. sapiens*; Mbr, *M. brevicollis*; Nve, *N. vectensis*.

Our database search did not identify the putative primordial CatSper sequence. It remains possible that the introduction of the primordial, probably distantly related, CatSper channel could be made through horizontal gene transfer between prokaryotes and primitive metazoan species, such as single 6-TMS domain Na_V_ channels Na_V_BP [19], NaChBac [61], and 11 bacterial NaChBac homologues [36]. All these bacterial Na_V_ channel homologues share a glutamate residue as the key acidic residue in the putative channel pore region [36], in contrast to the key aspartate residue conserved in CatSpers (Fig. 2). We analyzed 40 more bacterial protein sequences (protein cluster CLS1187052), which display high sequence homology and structural similarity to NaChBac. None of these 40 bacterial homologues grouped with CatSper sequences (Fig. 5). However, two (GI No. 56963529 and 134099759) have the key aspartate residue in the putative pore region, and it would be interesting compare their Ca^2+^-selectivity, voltage-dependence and kinetics with the CatSper channel.

### Rapid evolution of the CatSper channel complex

Reproductive system proteins are subjected to selective evolutionary forces and often undergo accelerated evolution and functional divergence [25]–[27]. Indeed, mammalian sperm-specific proteins, mostly cell surface binding proteins and enzymes, are rapidly evolving [26]. Sperm-specific channels and transporters have not been extensively studied, but positive selection on indel substitutions does occur in the first exon of the *CatSper1* gene in mammals [27], [62].

Non-synonymous (*d_N_*) and synonymous (*d_S_*) nucleotide substitution values as well as *d_N_*/*d_S_* ratios are often used for detecting evolutionary rates [63]. We calculated *d_N_* and *d_S_* values of human-mouse orthologous gene pairs of *CatSper* and *CatSerβ* genes, using the codeml program implemented in the PAML package [39]. The *d_N_* value of *CatSper* and *CatSerβ* genes (average = 0.268, Table 1) is 5.8- and 3.2-fold larger, respectively, than housekeeping genes and non-sperm tissue-specific genes [64], and 1.5 fold larger than other sperm-specific genes [26]. Thus, both CatSpers and CatSperβ appear to be subjected to high selective forces that promote amino acid diversity.

**Table 1:** *d_N_/d_S_* ratios between human and mouse *CatSper* and *CatSperβ* genes.

Full-length	*d_N_*	*d_S_*	*d_N_/d_S_*	TMS	*d_N_*	*d_S_*	*d_N_/d_S_*
*CatSper1*	0.353	1.358	0.260	*CatSper1*	0.096	1.547	0.062
*CatSper2*	0.226	0.698	0.324	*CatSper2*	0.1356	0.404	0.336
*CatSper3*	0.233	1.234	0.189	*CatSper3*	0.179	1.256	0.143
*CatSper4*	0.202	1.046	0.193	*CatSper4*	0.098	1.283	0.077
*CatSperβ*	0.328	0.801	0.409				
Average	0.268	1.027	0.275				
Ref. [68]	Means	Means	Means	Ref. [27]	Means	Means	Means
Housekeeping genes	0.046	0.447	0.093	Other tissue-specific genes	0.073	0.41	0.19
Tissue-specific genes	0.083	0.492	*0.172	Sperm-specific genes	0.18	0.45	0.50

* Calculated from 7 groups of tissue specific genes.

All data shown here are derived form comparison of human-mouse orthologs.

Abbreviation: *TMS*, transmembrane segments.

The *d_S_* values (Table 1) indicate that *CatSper* and *CatSerβ* genes contain high mutation rates, with an ∼2-fold increase over those of housekeeping genes, other tissue-specific genes and sperm-specific genes. Thus, *CatSper* and *CatSerβ* genes have an average *d_N_*/*d_S_* ratio that is smaller than other sperm-specific genes. Nevertheless, the average *d_N_*/*d_S_* ratio of *CatSper* and *CatSerβ* genes is ∼3- and 1.6-fold larger than housekeeping genes and other tissue-specific genes, respectively (Table 1). In addition, even though mammalian synonymous mutation rates (*d_S_* values) are generally considered selectively neutral, recent studies suggest that synonymous mutations might also be subjected to selection, possibly through their effects on splicing and/or mRNA stability [65]. Thus, *CatSper* and *CatSerβ* mRNA might also be processed, as shown in recent studies on human and mouse homologues of the cystic fibrosis transmembrane conductance regulator Cl^−^ channel [66]. Interestingly, *CatSperβ* has the highest *d_N_*/*d_S_* ratio of the genes examined (0.409; Table 1). The exact number of CatSperβ subunits in the channel complex is not yet known. Presumably, based on the observations of other tetrameric 6-TMS voltage-gated ion channels such as K^+^ channels [67], multiple CatSperβ proteins may be in the CatSper1-4 channel complex.

Rapid evolution of all four CatSper proteins appeared to have occurred at different domains in CatSpers. For instance, CatSper1 underwent strong positive selection in the N-terminal cytoplasmic domain [27], [62], but displayed evolutionary constraint in the 6-TMS domains (*d_N_*/*d_S_*, 0.062) (Table 1). CatSper 4 displays similar constraints on its TMS domains with a *d_N_*/*d_S_* value of 0.077. In contrast, the TMS domains of CatSper2 and CatSper3 were driven by higher selective forces (*d_N_*/*d_S_*, 0.336 and 0.143, respectively). The distinct evolutionary patterns in CatSper channel domains may promote channel diversity in modulating potential protein or second-messenger interactions [12]. CatSperβ, as perhaps the sole accessory protein, may have evolved even faster to accommodate concerted evolution at differential evolutionary sites of CatSper proteins, resulting in a high *d_N_*/*d_S_* ratio.

### Functional divergence of the CatSper protein family

The identification of many CatSper sequences, especially from invertebrates, allows us to calculate the functional divergence between CatSper1-4 (as shown previously [32], [68]). The coefficients of functional divergence between pairs of CatSper1-4 groups were estimated to be significant, ranging from 0.336±0.092 (CatSper3 vs. CatSper4) to 0.536±0.116 (CatSper2 vs. CatSper4) (Table S7). Thus, each CatSper group appears to have experienced altered evolutionary constraints after divergence from a putative ancestral duplication. Consistently, functional distance analysis also shows similarly long functional branch lengths (Fig. S1), implying that all four CatSpers diverged at comparable overall evolutionary rates from the putative primordial CatSper protein. Nevertheless, despite the similar overall evolutionary rates, the evolutionary processes might act differentially on specific sites that are unique in each CatSper gene (data not shown). Our data suggest that CatSper1-4 have acquired evolutionary novelties through substantial altered functional constraints after possible ancestral replication. Finally, rapid evolution of CatSpers (Table 1) still plays an important role in modulating CatSper functions in mammalian lineages.

In conclusion, we have carried out a comprehensive evolutionary genomics study of CatSper and CatSperβ proteins that constitutes the Ca^2+^ channel complex critical for sperm Ca^2+^ hyperactivation in mammals. With continued advances in genome biology, the evolutionary genomics approaches undertaken here will further illuminate the lineage-specific distribution of Ca^2+^ channels and transporters at the genomic scale and greatly facilitate deciphering the Ca^2+^ signaling codes in a species- and/or tissue-specific manner.

## Supporting Information

Figure S1 Tree topology of functional distance analysis of the CatSper protein family. Type I functional branch length bF was calculated as described in Materials and methods in Table S7. bF is an estimation of evolutionary distance of each CatSper group (1–4) to the putative primordial CatSper protein before replication (center circle).
(2.31 MB TIF)

Table S1 List of CatSper Proteins Used for Analyses
(0.31 MB PDF)

Table S2 Genome Synteny - CatSperβ
(0.07 MB PDF)

Table S3 Genome Synteny - CatSper1
(0.06 MB PDF)

Table S4 Genome Synteny - CatSper2
(0.07 MB PDF)

Table S5 Genome Synteny - CatSper3
(0.06 MB PDF)

Table S6 Genome Synteny - CatSper4
(0.06 MB PDF)

Table S7 (0.03 MB PDF)
