# Supplementary figures and images for: Evolutionary Genomics Reveals Lineage-Specific Gene Loss and Rapid Evolution of a Sperm-Specific Ion Channel Complex: CatSpers and CatSperβ
Source: PLoS One. 2008 Oct 30;3(10):e3569. doi: 10.1371/journal.pone.0003569 (PMC2572835; doi:10.1371/journal.pone.0003569)

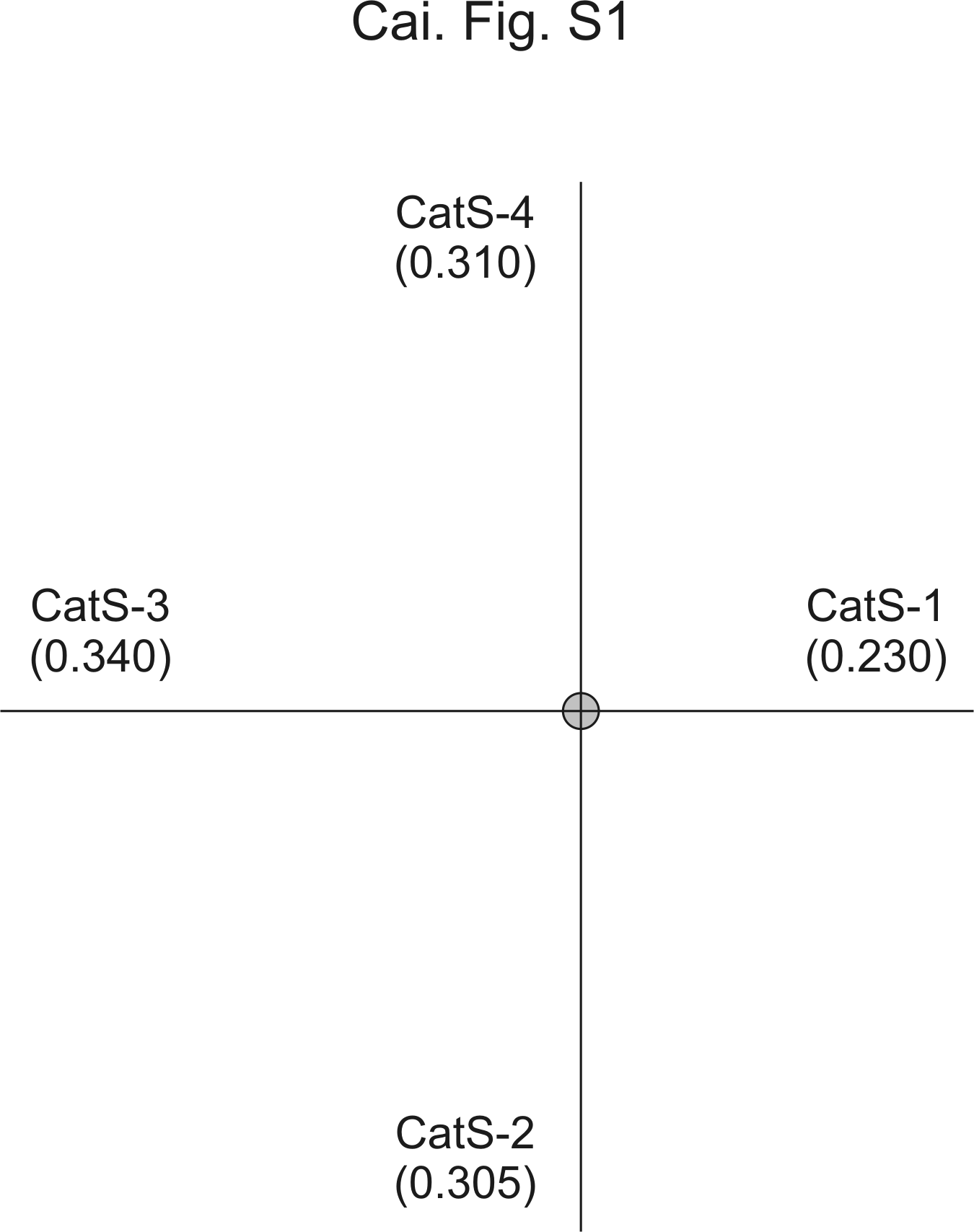

Supplement: Figure S1 — Tree topology of functional distance analysis of the CatSper protein family. Type I functional branch length bF was calculated as described in Materials and methods in Table S7. bF is an estimation of evolutionary distance of each CatSper group (1–4) to the putative primordial CatSper protein before replication (center circle). (2.31 MB TIF) [file pone.0003569.s001.tif]
