# Supplementary material for: Evolutionary Genomics Reveals Lineage-Specific Gene Loss and Rapid Evolution of a Sperm-Specific Ion Channel Complex: CatSpers and CatSperβ
Source: PLoS One. 2008 Oct 30;3(10):e3569. doi: 10.1371/journal.pone.0003569 (PMC2572835; doi:10.1371/journal.pone.0003569)
Supplement: Table S1 — List of CatSper Proteins Used for Analyses (0.31 MB PDF) [file pone.0003569.s002.pdf]

Table S1. List of CatSper Proteins Used for Analyses

Name	Organism	Common Name	Identifier	Database
		Invertebrates - Cnidarians		
NveCatS-1	*Nematostella vectensis*	starlet sea anemone	XP_001641973.1	GenBank
NveCatS-2	*Nematostella vectensis*	starlet sea anemone	XP_001629309.1	GenBank
*NveCatS-3	*Nematostella vectensis*	starlet sea anemone	XP_001629965.1	GenBank
*NveCatS-4	*Nematostella vectensis*	starlet sea anemone	XP_001631669.1	GenBank
NveCatS-β	*Nematostella vectensis*	starlet sea anemone	EDO49139.1	GenBank
		Invertebrates – Ctenophora		
PpiCatS-1	*Pleurobrachia pileus*	sea gooseberry	CU423497.1 (nt)	GenBank
PpiCatS-2	*Pleurobrachia pileus*	sea gooseberry	CU417395.1 (nt)<br>CU422181.1 (nt)	GenBank
		Invertebrates - Echinoderms		
SpuCatS-1	*Strongylocentrotus purpuratus*	Purple Sea Urchin	NW_001347864.1 (nt)	GenBank
SpuCatS-2	*Strongylocentrotus purpuratus*	Purple Sea Urchin	XP_001193971.1	GenBank
SpuCatS-3	*Strongylocentrotus purpuratus*	Purple Sea Urchin	NW_001320541.1 (nt)	GenBank
SpuCatS-4	*Strongylocentrotus purpuratus*	Purple Sea Urchin	NW_001312374.1 (nt)	GenBank
*SpuCatS-β	*Strongylocentrotus purpuratus*	Purple Sea Urchin	|NW_001356100.1 (nt)	GenBank
		Invertebrates – Cephalochordates (Amphioxus)		
BflCatS-1	*Branchiostoma floridae*	Florida Lancelet	scaffold_42 (nt)	JGI
BflCatS-2	*Branchiostoma floridae*	Florida Lncelet	scaffold_346 (nt)	JGI
BflCatS-3	*Branchiostoma floridae*	Florida Lancelet	scaffold_92 (nt)	JGI
BflCatS-4	*Branchiostoma floridae*	Florida Lancelet	scaffold_135 (nt)	JGI
*BflCatS-β	*Branchiostoma floridae*	Florida Lancelet	scaffold_62 (nt)	JGI
		Invertebrates – Urochodates (Tunicates)		
CsaCatS-1	*Ciona savignyi*	Sea Squirt	Reftig_44 (nt)	Ensembl
CsaCatS-2	*Ciona savignyi*	Sea Squirt	Reftig_464 (nt)	Ensembl
CsaCatS-3	*Ciona savignyi*	Sea Squirt	Reftig_19 (nt)	Ensembl
CsaCatS-4	*Ciona savignyi*	Sea Squirt	Reftig_17 (nt)	Ensembl
*CsaCatS-β	*Ciona savignyi*	Sea Squirt	Reftig_4 (nt)	Ensembl
CinCatS-1	*Ciona intestinalis*	Sea Squirt	Cioin2: 221463	JGI
CinCatS-2	*Ciona intestinalis*	Sea Squirt	Chunk scaffold_215 (nt)	JGI
*CinCatS-3	*Ciona intestinalis*	Sea Squirt	Chunk 2q_165 (nt)	JGI
CinCatS-4	*Ciona intestinalis*	Sea Squirt	Chunk 2q_460 (nt)	JGI
CinCatS-β	*Ciona intestinalis*	Sea Squirt	Chunk 9q_416 (nt)	JGI
		Vertebrates – Chondrichthyes (Cartilaginous Fishes)		
*CmiCatS-2	*Callorhinchus milii*	Elephant Shark	AAVX01047343.1 (nt)	GenBank
*CmiCatS-3	*Callorhinchus milii*	Elephant Shark	AAVX01241861.1 (nt)	GenBank
*CmiCatS-4	*Callorhinchus milii*	Elephant Shark	AAVX01198782.1 (nt)<br>AAVX01295261.1 (nt)<br>AAVX01583470.1 (nt)<br>AAVX01099504.1 (nt)	GenBank
*CmiCatS-β	*Callorhinchus milii*	Elephant Shark	AAVX01468406.1 (nt)<br>AAVX01191566.1 (nt)<br>AAVX01360901.1 (nt)<br>AAVX01416650.1 (nt)	GenBank
*LerCatS-2	*Leucoraja erinacea*	Little Skate	CO050315.1 (nt)	GenBank
		Vertebrates – Reptiles		
AcaCatS-1	*Anolis carolinensis*	Anole Lizard	GENSCAN00000056132	Ensembl
AcaCatS-2	*Anolis carolinensis*	Anole Lizard	GENSCAN00000067675	Ensembl
*AcaCatS-3	*Anolis carolinensis*	Anole Lizard	GENSCAN00000120241	Ensembl
*AcaCatS-4	*Anolis carolinensis*	Anole Lizard	GENSCAN00000121458	Ensembl
AcaCatS-β	*Anolis carolinensis*	Anole Lizard	GENSCAN00000081873	Ensembl
		Vertebrates – Mammals		
MusCatS-1	*Mus musculus*	House Mouse	NP_647462.1	GenBank

MusCatS-2	*Mus musculus*	House Mouse	NP_694715.2	GenBank
MusCatS-3	*Mus musculus*	House Mouse	Q80W99.2	GenBank
MusCatS-4	*Mus musculus*	House Mouse	NP_808534.1	GenBank
MusCatS-β	*Mus musculus*	House Mouse	NP_766611.1	GenBank
RnoCatS-1	*Rattus norvegicus*	Norway rat	XP_001070492.1	GenBank
RnoCatS-2	*Rattus norvegicus*	Norway rat	NP_001012220.1	GenBank
RnoCatS-3	*Rattus norvegicus*	Norway rat	NP_001099571.1	GenBank
RnoCatS-4	*Rattus norvegicus*	Norway rat	XP_001066739.1	GenBank
RnoCatS-β	*Rattus norvegicus*	Norway rat	XP_001065501.1	GenBank
MdoCatS-1	*Monodelphis domestica*	Gray Short-tailed Opossum	XP_001379458.1	GenBank
MdoCatS-2	*Monodelphis domestica*	Gray Short-tailed Opossum	NW_001581835.1 (nt)	GenBank
MdoCatS-3	*Monodelphis domestica*	Gray Short-tailed Opossum	XP_001375484.1	GenBank
MdoCatS-4	*Monodelphis domestica*	Gray Short-tailed Opossum	XP_001369666.1	GenBank
MdoCatS-β	*Monodelphis domestica*	Gray Short-tailed Opossum	XP_001369855.1	GenBank
OanCatS-1	*Ornithorhynchus anatinus*	Platypus	NW_001630702.1 (nt)	GenBank
OanCatS-2	*Ornithorhynchus anatinus*	Platypus	XP_001521786.1	GenBank
OanCastS-3	*Ornithorhynchus anatinus*	Platypus	NW_001748254.1 (nt)	GenBank
OanCatS-4	*Ornithorhynchus anatinus*	Platypus	XP_001518227.1	GenBank
OanCatS-β	*Ornithorhynchus anatinus*	Platypus	XP_001514501.1	GenBank
EcaCatS-1	*Equus caballus*	Horse	XP_001491183.1	GenBank
EcaCatS-2	*Equus caballus*	Horse	NW_001799682.1 (nt)	GenBank
EcaCatS-3	*Equus caballus*	Horse	XP_001502783.1	GenBank
EcaCatS-4	*Equus caballus*	Horse	NW_001799705.1	GenBank
EcaCatS-β	*Equus caballus*	Horse	XP_001494623.1	GenBank
BtaCatS-1	*Bos taurus*	Cattle	XP_601857.3	GenBank
BtaCatS-2	*Bos taurus*	Cattle	XP_595874.3	GenBank
BtaCatS-3	*Bos taurus*	Cattle	XP_613302.2	GenBank
BtaCatS-4	*Bos taurus*	Cattle	XP_599038.3	GenBank
BtaCatS-β	*Bos taurus*	Cattle	NW_001494053.1 (nt)	GenBank
CfaCatS-1	*Canis familiaris*	Dog	XP_533225.2	GenBank
CfaCatS-2	*Canis familiaris*	Dog	XP_851279.1	GenBank
CfaCatS-3	*Canis familiaris*	Dog	XP_538633.2	GenBank
CfaCatS-4	*Canis familiaris*	Dog	XP_544484.2	GenBank
CfaCatS-β	*Canis familiaris*	Dog	XP_848876.1	GenBank
*ScrCatS-4	*Sus scrofa*	Pig	CAQ34765.1	GenBank
CpoCatS-β	*Cavia porcellus*	Guinea Pig	AAKN02025631.1 (nt)	GenBank

Vertebrates – Mammals (Primates)

MmuCatS-1	*Macaca mulatta*	Rhesus Monkey	XP_001118063.1	GenBank
MmuCatS-2	*Macaca mulatta*	Rhesus Monkey	XP_001108563.1	GenBank
MmuCatS-3	*Macaca mulatta*	Rhesus Monkey	XP_001103397.1	GenBank
MmuCatS-4	*Macaca mulatta*	Rhesus Monkey	XP_001108634.1	GenBank
*MmuCatS-β	*Macaca mulatta*	Rhesus Monkey	BAE00646.1	GenBank
PtrCatS-1	*Pan troglodytes*	Chimpanzee	NW_001222291.1 (nt)	GenBank
PtrCatS-2	*Pan troglodytes*	Chimpanzee	XP_001159071.1	GenBank
PtrCatS-3	*Pan troglodytes*	Chimpanzee	XP_527015.2	GenBank
PtrCatS-4	*Pan troglodytes*	Chimpanzee	XP_001139244.1	GenBank
PtrCatS-β	*Pan troglodytes*	Chimpanzee	XP_001144834.1	GenBank
HsaCatS-1	*Homo sapiens*	Human	NP_444282.2	GenBank
HsaCatS-2	*Homo sapiens*	Human	Q96P56.2	GenBank
HsaCatS-3	*Homo sapiens*	Human	NP_821138.1	GenBank
HsaCatS-4	*Homo sapiens*	Human	NP_937770.1	GenBank
HsaCatS-β	*Homo sapiens*	Human	NP_079040.2	GenBank
*AgeCatS-1	*Ateles geoffroyi*	Black-handed Spider Monkey	AAQ95774.1	GenBank
*AtrCatS-1	*Aotus trivirgatus*	Douroucouli	AAQ95776.1	GenBank
*CaeCatS-1	*Cercopithecus aethiops*	African Green Monkey	AAQ95780.1	GenBank

*CguCatS-1	*Colobus guereza*	Guereza	AAQ95782.1	GenBank
*GgoCatS-1	*Gorilla gorilla*	Western Gorilla	AAQ95786.1	GenBank
*LcaCatS-1	*Lemur catta*	Ring-tailed lemur	AAQ95788.1	GenBank
*LlaCatS-1	*Lagothrix lagotricha*	Common Woolly Monkey	AAQ95773.1	GenBank
*MtaCatS-1	*Miopithecus talapoin*	talapoin	AAQ95778.1	GenBank
*PhaCatS-1	*Papio hamadryas*	Hamadryas Baboon	AAQ95781.1	GenBank
*PpaCatS-1	*Pan paniscus*	Pygmy Chimpanzee	AAQ95784.1	GenBank
*PpyCatS-1	*Pongo pygmaeus*	Bornean Orangutan	AAQ95787.1	GenBank
*SoeCatS-1	*Saguinus oedipus*	Cotton-top Tamarin	AAQ95775.1	GenBank
*SscCatS-1	*Saimiri sciureus*	Common Squirrel Monkey	AAQ95777.1	GenBank
CjaCatS-2	*Callithrix jacchus*	white-tufted-ear marmoset	ABY84144.1	GenBank
*MfaCatS-2	*Macaca fascicularis*	Crab-eating Macaque	BAE01081.1	GenBank
PanCatS-2	*Papio anubis*	Olive Baboon	ABY40813.1	GenBank
*MfaCatS-3	*Macaca fascicularis*	Crab-eating Macaque	XP_001118991.1	GenBank

NOTE — Naming of sequences: we describe all CatSper and CatSper-β proteins in this study using an abbreviation of genus and species name, followed by names after their phylogenetic relationship with characterized human counterparts. Abbreviations: *nt*, nucleotides.

Database: GenBank, National Center for Biotechnology Information, http://www.ncbi.nlm.nih.gov/Genbank/; Ensembl, http://www.ensembl.org/; JGI, Joint Genome Institute, http:// www.jgi.doe.gov/.

*Sequences that failed in the chi-square test in Tree-Puzzle or contained more than 15% gaps in the refined alignments.
