# Supplementary material for: Evolutionary Genomics Reveals Lineage-Specific Gene Loss and Rapid Evolution of a Sperm-Specific Ion Channel Complex: CatSpers and CatSperβ
Source: PLoS One. 2008 Oct 30;3(10):e3569. doi: 10.1371/journal.pone.0003569 (PMC2572835; doi:10.1371/journal.pone.0003569)
Supplement: Table S2 — Genome Synteny - CatSperβ (0.07 MB PDF) [file pone.0003569.s003.pdf]

Table S2. Genome Synteny – CatSper-β

Genes	*RPS6KA5*	*C14orf159*	*GPR68*	*CCDC88C*	*SMEK1*	***CatSper-β***	*TC2N*	*FBLN5*	*TRIP11*	*ATXN3*	*NDUFB1*
HsaCh14	+	+	+	+	+	+	+	+	+	+	+
MusChr12	+	+	+	+	+	+	+	+	+	+	+
GgaCh5	+	+	+	+	+	- (fragment)	+	+	+	+	+

RPS6KA5, ribosomal protein S6 kinase, 90kDa, polypeptide 5
C14orf159, chromosome 14 open reading frame 159;
GPR68, G protein-coupled receptor 68
CCDC88C, coiled-coil domain containing 88C;
SMEK1, SMEK homolog 1, suppressor of mek1 (Dictyostelium);

TC2N, tandem C2 domains, nuclear;
FBLN5, fibulin 5;
TRIP11, thyroid hormone receptor interactor 11
ATXN3, ataxin 3;
NDUFB1, NADH dehydrogenase (ubiquinone) 1 beta subcomplex, 1, 7kDa;

Hsa, *H. sapiens*; Mus, *M. musculus*; Gga, *G. gallus*;
Ch - chromosome
(?) – Not mapped to chromosomes.
