# Supplementary material for: Evolutionary Genomics Reveals Lineage-Specific Gene Loss and Rapid Evolution of a Sperm-Specific Ion Channel Complex: CatSpers and CatSperβ
Source: PLoS One. 2008 Oct 30;3(10):e3569. doi: 10.1371/journal.pone.0003569 (PMC2572835; doi:10.1371/journal.pone.0003569)
Supplement: Table S3 — Genome Synteny - CatSper1 (0.06 MB PDF) [file pone.0003569.s004.pdf]

Table S3. Genome Synteny – CatSper-1

No obvious synteny between human and mouse chromosomal regions containing CatSper1 and the chicken genome

Genes	*TSGA10IP*	*SART1*	*EIF1AD*	*BANF1*	*CST6*	***CatSper1***	*LOC100131232*	*GAL3ST3*	*SF3B2*	*PACS1*	*KLC2*
HsaCh11	+	+	+	+	+	+	+	+	+	+	+
MusCh9	+	+	+	+	+	+	+	+	+	+	+
GgaCh	-	(?)	-	(?)	-	-	-	-	-	+<br>Ch3	+<br>Ch3

TSGA10IP, testis specific, 10 interacting protein;
SART1, squamous cell carcinoma antigen recognized by T cells;
EIF1AD, eukaryotic translation initiation factor 1A domain containing;
BANF1, barrier to autointegration factor 1;
CST6, cystatin E/M;

LOC100131232, hypothetical LOC100131232;
GAL3ST3, galactose-3-O-sulfotransferase 3;
SF3B2 ,splicing factor 3b, subunit 2, 145kDa;
PACS1, phosphofurin acidic cluster sorting protein 1;
KLC2, kinesin light chain 2;

Hsa, *H. sapiens*; Mus, *M. musculus*; Gga, *G. gallus*;
Ch - chromosome.
