# Supplementary material for: Evolutionary Genomics Reveals Lineage-Specific Gene Loss and Rapid Evolution of a Sperm-Specific Ion Channel Complex: CatSpers and CatSperβ
Source: PLoS One. 2008 Oct 30;3(10):e3569. doi: 10.1371/journal.pone.0003569 (PMC2572835; doi:10.1371/journal.pone.0003569)
Supplement: Table S4 — Genome Synteny - CatSper2 (0.07 MB PDF) [file pone.0003569.s005.pdf]

Table S4. Genome Synteny – CatSper-2

Genes	*TUBG CP4*	*TP53B P1*	*MA P1A*	*HISPP D2A*	*CKMT 1B*	*STRC*	***CatSper-2***	*HISPP D2B*	*CKMT 1A*	*STRCP*	*CATSP ER2P1*	*PDIA3*	*ELL3*	*SERF2*	*SERIN C4*
HsaCh15	+	+	+	+	+	+	+	+	+	+	+	+	+	+	+
								(primate-specific duplication)							
MusCh2	+	+	+	+	+	+	+					+	+	+	+
GgaCh10	+	+	+	+	+	-	- (fragment)					+		+	+

TUBGCP4, tubulin, gamma complex associated protein 4;
TP53BP1, tumor protein p53 binding protein 1;
MAP1A, microtubule-associated protein 1A;
HISPPD2A, histidine acid phosphatase domain containing 2B (*pseudogene*);
CKMT1B, creatine kinase, mitochondrial 1B;
STRC, stereocilin;

HISPPD2B, histidine acid phosphatase domain containing 2B (*pseudogene*);
CKMT1A, creatine kinase, mitochondrial 1A;
STRCP, stereocilin pseudogene;
CATSPER2P1, cation channel, sperm associated 2 (*pseudogene*);
PDIA3, protein disulfide isomerase family A, member 3;
ELL3, elongation factor RNA polymerase II-like 3;
SERF2, small EDRK-rich factor 2;
SERINC4, serine incorporator 4;

Hsa, *H. sapiens*; Mus, *M. musculus*; Gga, *G. gallus*;
Ch – chromosome.
