# Supplementary material for: Evolutionary Genomics Reveals Lineage-Specific Gene Loss and Rapid Evolution of a Sperm-Specific Ion Channel Complex: CatSpers and CatSperβ
Source: PLoS One. 2008 Oct 30;3(10):e3569. doi: 10.1371/journal.pone.0003569 (PMC2572835; doi:10.1371/journal.pone.0003569)
Supplement: Table S5 — Genome Synteny - CatSper3 (0.06 MB PDF) [file pone.0003569.s006.pdf]

Table S5. Genome Synteny – CatSper-3

Genes	*LOC153328*	*CXCL14*	*NEUROG1*	*H2AFY*	*PITX1*	***CatSper3***	*PCBD2*	*TXNDC15*	*C5orf24*	*DDX46*	*CAMLG*
HsaCh5	+	+	+	+	+	+	+	+	+	+	+
MusCh13	+	+	+	+	+	+	+	+	+	+	+
GgaCh13	+	+	+	+	+	- (fragment)	+	+	+	+	+

LOC153328, LOC153328 similar to CG4995 gene product;
CXCL14, chemokine (C-X-C motif) ligand 14;
NEUROG1, neurogenin 1;
H2AFY, H2A histone family, member Y;
PITX1, paired-like homeodomain transcription factor 1;

PCBD2, pterin-4 alpha-carbinolamine dehydratase/dimerization cofactor of hepatocyte nuclear factor 1 alpha (TCF1) 2;
TXNDC15, thioredoxin domain containing 15;
C5orf24, chromosome 5 open reading frame 24;
DDX46, DEAD (Asp-Glu-Ala-Asp) box polypeptide 46;
CAMLG, calcium modulating ligand;

Hsa, *H. sapiens*; Mus, *M. musculus*; Gga, *G. gallus*;
Ch – chromosome.
