# Supplementary material for: Evolutionary Genomics Reveals Lineage-Specific Gene Loss and Rapid Evolution of a Sperm-Specific Ion Channel Complex: CatSpers and CatSperβ
Source: PLoS One. 2008 Oct 30;3(10):e3569. doi: 10.1371/journal.pone.0003569 (PMC2572835; doi:10.1371/journal.pone.0003569)
Supplement: Table S6 — Genome Synteny - CatSper4 (0.06 MB PDF) [file pone.0003569.s007.pdf]

Table S6. Genome Synteny – CatSper-4

Genes	*TRIM63*	*PDIK1L*	*GRRP1*	*ZNF593*	*CNKSR1*	***CATSPER4***	*EG665186*	*Ccdc21*	*Sh3bgrl3*	*Ubxd5*	*Aim1l*
HsaCh1	+	+	+	+	+	+	+	+	+	+	+
MusCh4	+	+	+	+	+	+	+	+	+	+	+
GgaCh23	+	+	+	+	+	-	-	+	+	+	+

TRIM63, tripartite motif-containing 63;
PDIK1L, PDLIM1 interacting kinase 1 like;
GRRP1, glycine/arginine rich protein 1;
ZNF593, zinc finger protein 593;
CNKSR1, connector enhancer of kinase suppressor of Ras 1;

EG665186, predicted gene, EG665186;
Ccdc21, coiled-coil domain containing 21;
Sh3bgrl3, SH3 domain binding glutamic acid-rich protein-like 3;
Ubxd5, UBX domain containing 5;
Aim1l, absent in melanoma 1-like;

Hsa, *H. sapiens*; Mus, *M. musculus*; Gga, *G. gallus*;
Ch – chromosome.
