# Supplementary material for: Evolutionary Genomics Reveals Lineage-Specific Gene Loss and Rapid Evolution of a Sperm-Specific Ion Channel Complex: CatSpers and CatSperβ
Source: PLoS One. 2008 Oct 30;3(10):e3569. doi: 10.1371/journal.pone.0003569 (PMC2572835; doi:10.1371/journal.pone.0003569)
Supplement: Table S7 — (0.03 MB PDF) [file pone.0003569.s008.pdf]

Table S7

Materials and methods

Functional divergence and functional distance analyses

Site-specific evolutionary rates after gene duplication (type I functional divergence) may differ in the protein family, possibly due to altered functional constraints [1]. Maximum likelihood estimate for θ, the coefficient of type I functional divergence, was measured with the program DIVERGE (version 2.0) [2]. A *de novo* neighbor-joining tree was constructed with Poisson distance and re-rooted. Four clusters of the phylogenetic tree, each corresponding to one of the four CatSper groups, were then selected for likelihood ratio tests analysis.

After the coefficient of type I functional divergence for each pair of clusters was calculated, the functional branch length for each cluster, b_F, was estimated to indicate the degree of altered functional constraints [2]. The value of b_F is related to the evolutionary rates of duplicated genes compared with the ancestral gene. $b_F = 0$ suggests the site-specific evolutionary rates of the duplicated gene nearly identical to those of the ancestral gene.

Table S2. Functional divergence between CatSper protein subfamilies

*θ	CatS-2	CatS-3	CatS-4
CatS-1	0.525 ± 0.129	0.433 ± 0.116	0.483 ± 0.146
CatS-2	\	0.408 ± 0.076	0.536 ± 0.116
CatS-3	\	\	0.336 ± 0.092

*θ - Coefficients of type I functional divergence, all $p < 0.05$.

References:

[1] X. Gu, Maximum-likelihood approach for gene family evolution under functional divergence, Mol. Biol. Evol. 18 (2001) 453-64.

[2] X. Gu, K. Vander Velden, DIVERGE: phylogeny-based analysis for functional-structural divergence of a protein family, Bioinformatics 18 (2002) 500-1.
